# Presence of Tumor Necrosis Factor-Alpha in Urine Samples of Patients With Chronic Low Back Pain Undergoing Chiropractic Care: Preliminary Findings From a Prospective Cohort Study

**DOI:** 10.3389/fnint.2022.879083

**Published:** 2022-04-12

**Authors:** Carlos Gevers-Montoro, Mar Romero-Santiago, Lisa Losapio, Francisco Miguel Conesa-Buendía, Dave Newell, Luis Álvarez-Galovich, Mathieu Piché, Arantxa Ortega-De Mues

**Affiliations:** ^1^Madrid College of Chiropractic – RCU María Cristina, Madrid, Spain; ^2^Department of Anatomy, Université du Québec à Trois-Rivières, Trois-Rivières, QC, Canada; ^3^CogNAC Research Group, Université du Québec à Trois-Rivières, Trois-Rivières, QC, Canada; ^4^Instituto de Investigación Sanitaria, Fundación Jiménez-Díaz, Madrid, Spain; ^5^Chiropractic Department, AECC University College, Bournemouth, United Kingdom

**Keywords:** low back pain, inflammatory cytokines, urinalysis, tumor necrosis factor alpha, chronic pain, chiropractic

## Abstract

**Background and aims:**

Low back pain is the leading cause of years lived with disability worldwide. Chiropractors employ different interventions to treat low back pain, including spinal manipulative therapy, although the mechanisms through which chiropractic care improves low back pain are still unclear. Clinical research and animal models suggest that spinal manipulation might modulate plasma levels of inflammatory cytokines, which have been involved in different stages of low back pain. More specifically, serum levels of Tumor Necrosis Factor-alpha (TNF-α) have been found to be elevated in patients with chronic low back pain. We aimed to investigate whether urine from chronic low back pain patients could be an appropriate medium to measure concentrations of TNF-α and to examine possible changes in its levels associated to chiropractic care.

**Methods:**

Urine samples were collected from 24 patients with chronic low back pain and TNF-α levels were analyzed by ELISA before and after 4–6 weeks of care compared to a reference value obtained from 5 healthy control subjects, by means of a Welch’s *t*-test. Simultaneously, pain intensity and disability were also evaluated before and after care. Paired *t*-tests were used to compare mean pre and post urinary concentrations of TNF-α and clinical outcomes.

**Results:**

Significantly higher baseline levels of urinary TNF-α were observed in chronic low back pain patients when compared to our reference value (*p* < 0.001), which were significantly lower after the period of chiropractic treatment (*p* = 0.03). Moreover, these changes were accompanied by a significant reduction in pain and disability (both *p* < 0.001). However, levels of urinary TNF-α were not correlated with pain intensity nor disability.

**Conclusion:**

These results suggest that urine could be a good milieu to assess TNF-α changes, with potential clinical implications for the management of chronic low back pain.

## Introduction

Low back pain (LBP) is a common condition that is currently considered to be the first cause of years lived with disability worldwide, impacting most adults at least once in their lifetime (Hartvigsen et al., 2018). LBP has been the focus of recent reviews calling for action upon it as a critical public health issue with huge economic implications, mostly related to the loss of workdays and healthcare expenditure (Hartvigsen et al., 2018; Vlaeyen et al., 2018).

Most cases of LBP are described as being non-specific, implying that the etiology of the patient’s complaints is unknown and cannot be attributed to a single tissue or pathology (Vlaeyen et al., 2018). A growing body of research is being directed toward identifying biomarkers and biopsychosocial risk factors that influence the course of the disease (Vlaeyen et al., 2018). A variety of inflammatory cytokines profiles and biochemical markers have been linked to different stages of non-specific LBP (Li et al., 2016; Wang et al., 2016; Klyne et al., 2017; James et al., 2018b; Teodorczyk-Injeyan et al., 2019; Lim et al., 2020; Morris et al., 2020). These cytokine profiles were predominantly quantified through serum analysis (Li et al., 2016; Wang et al., 2016; Klyne et al., 2017), although accumulating evidence suggests that it may be possible to detect some of these biomarkers in urine with a high degree of correlation with circulating levels (Moldawer, 1997; Sirera et al., 2003; Prasad et al., 2016). Thus, urinalysis could provide a cheaper, more feasible alternative to blood samples that is much easier to collect in a private or smaller clinical setting.

Recent studies have reported an important role of Tumor Necrosis Factor-alpha (TNF-α) in the pathogenesis and in possible treatment strategies of chronic LBP (CLBP) (Wang et al., 2008, 2016; Sainoh et al., 2016; Lim et al., 2020). TNF-α is a potent pro-inflammatory cytokine that was closely associated with intervertebral disk degeneration, though it may also induce a downstream cascade of other cytokines involved in CLBP such as interleukin-6 or interleukin-1β (Risbud and Shapiro, 2014; Lim et al., 2020). Observational studies have found elevated serum levels of TNF-α in patients with severe sciatica and CLBP compared to patients with milder symptoms or healthy controls (Wang et al., 2008, 2016). The *in vitro* production of TNF-α was found to be increased (and positively correlated to that of interleukin 1β) in CLBP when compared to healthy control and acute LBP individuals (Teodorczyk-Injeyan et al., 2019). Animal studies have also reported an increased expression of TNF-α in multiple tissues during early chronic stages of intervertebral disk degeneration (James et al., 2018b).

Chiropractic care offers a conservative option for the management of CLBP. To address this condition, chiropractors use a multimodal approach, including manual therapy along with exercise and patient education as part of a toolkit of routine interventions (Walker et al., 2011; Beliveau et al., 2017). However, spinal manipulative therapy (SMT) is the main intervention utilized in chiropractic practice (Beliveau et al., 2017). SMT has been found to be as effective for CLBP as other recommended interventions, such as exercise therapy (Rubinstein et al., 2019; Gevers-Montoro et al., 2021b) and is currently recommended in the latest clinical practice guidelines (Qaseem et al., 2017; Wong et al., 2017). For both manipulative and exercise therapy, multiple potential mechanisms for pain relief have been proposed (Gevers-Montoro et al., 2021a; Wun et al., 2021). The specific mechanisms are still unknown, but human and animal studies have shown a correlation between SMT and a decrease in serum levels and *in vitro* production of pro-inflammatory mediators, including TNF-α (Teodorczyk-Injeyan et al., 2006, 2018, 2021; Roy et al., 2010; Song et al., 2016). Similarly, investigations on the mechanisms of exercise therapy for CLBP have also found that a reduction in levels of the same cytokine network may be involved in pain relief associated to exercise therapy (Cheng et al., 2015; Leung et al., 2016; James et al., 2018a).

Therefore, our objectives were to examine the possible presence of TNF-α in urine samples of CLBP patients, and to assess whether levels in this cytokine would vary significantly after a period of multimodal chiropractic care mainly based in SMT, in parallel to pain intensity and disability. We hypothesized that the urinary levels of TNF-α would be elevated in patients with CLBP compared to healthy participants. Additionally, we posited that urinary levels of this cytokine would change following a period of chiropractic care.

## Materials and Methods

### Ethical Approval

This study was conducted as a prospective cohort study conducted at the Madrid College of Chiropractic Outpatient Clinic (will be referred to as “the Clinic” from here on) in San Lorenzo de El Escorial, Spain. All experimental procedures conformed to the standards set by the latest revision of the Declaration of Helsinki and ethical approval was granted by the Madrid College of Chiropractic Research Ethics subcommittee before starting recruitment.

### Participant Recruitment

All participants in this study were recruited from the patient population of the Clinic upon initial evaluation by one of the investigators who supervised the patient recruitment. Any patient presenting for care with CLBP was screened for inclusion and exclusion criteria and all deemed eligible were offered to participate in the study. Patients who accepted signed specific informed consent forms for the study before entering the protocol. Patients were to be recruited with the following inclusion criteria: being between 18 and 75 years of age and a clinical presentation of chronic non-specific LBP (persistent or recurrent pain affecting any region between the lower margin of the 12th rib to the lower gluteal folds with or without referring to one or both lower limbs >12 weeks) (Vlaeyen et al., 2018). Patients were excluded if a specific pathology was suspected as the cause for their LBP and according to the established exclusion criteria presented in Table 1. These criteria were assessed during the case history and physical examination following routine procedures of the Clinic.

**TABLE 1:** Exclusion criteria.

✓ Suspected serious spinal pathology (e.g., fracture, inflammatory/infections spinal disease, cauda equina syndrome, malignancy, metastasis, unexplained weight loss or fever, neurological disorder)
✓ Specific pathology that cause back pain, such as internal organ disease, active inflammatory spondyloarthropathies, recent spinal trauma or surgery
✓ Presence of major pain or injury to other body regions in the previous 12 months
✓ Pregnancy
✓ Renal pathologies that can alter creatinine and urinary cytokines levels
✓ Sleep disorders (such as sleep apnea and snoring)
✓ Known depression, anxiety or other psychological disorders

In parallel, a sample of healthy volunteer participants without LBP or any other health concern was recruited. In this case, the only inclusion criterion was to be between 18 and 75 years of age, and the main exclusion criteria were the existence of any acute or chronic health condition, particularly if it could influence or modify inflammatory status. This included having experienced or having sought medical care for LBP in the last year, and having received SMT in the previous 6 months. Participants included in the control group signed an informed consent before urine sample collection.

### Treatment Protocol

Patients participating in the study underwent a customized chiropractic care plan consisting in each encounter of a case history, physical assessment and multimodal care (Walker et al., 2011), the latter starting on their second visit. This was based on full-spine SMT, exercise recommendations and patient education, with a patient-centered approach. All patients received full-spine SMT, however, all other interventions were used less frequently, based on patient and clinician preferences and needs. This pragmatic setting was preferred as it reflects better real clinical practice. Spinal manipulation consisted in high-velocity low amplitude thrust manipulations, applied to joints with restricted motion by hand with or without the assistance of a drop-piece mechanism. When used, exercise recommendations were based on a customized set of stretching and mobilization exercises focused on the lumbopelvic area, to be performed for less than 5 min on a daily basis. Patient education was limited to advice to remain active and reassurance about the benign nature of the patients’ condition. Care was delivered by a chiropractic intern, under the supervision of a trained chiropractor with at least 5 years of clinical expertise. The duration of care and the total number of treatment sessions received per patients was not pre-established. Instead, patients were offered a treatment plan adapted to their clinical presentation, with 1 or 2 weekly visits for a range of 4 to 8 weeks. This is in accordance with recommendations from a clinical practice guideline that suggests 1–3 weekly visits for about 4 weeks for CLBP (Globe et al., 2016).

At the end of the treatment period, a complete physical re-evaluation of the patient was performed, including evaluation of the main outcome measures, described in the following sections. Care was purposely not modified for the sake of the study. The control group did not receive any type of treatment. The inclusion of this group aimed at defining a reference value for urinary TNF-α in a healthy population, in order to confirm that persons with CLBP have higher than normal urinary TNF-α levels. TNF-α levels are presumed to remain relatively stable, nearing zero in the absence of active or chronic inflammation (Feghali and Wright, 1997; Biancotto et al., 2013; Wang et al., 2016). Reference ranges and biological variability over 6 weeks for serum TNF-α in healthy subjects have been reported (Todd et al., 2013). A mean value of 1.51 pg/mL and the upper limits of 2.53 pg/ml (95th percentile) and 3.30 pg/mL (99th percentile) were deemed acceptable for diagnosis of systemic inflammation (Todd et al., 2013). To our knowledge, no reference values for urinary levels of TNF-α have been published to date. Therefore, the control group served as a comparison group providing a reference value, which would be used to test the hypothesis of abnormally high levels of TNF-α in the CLBP groups when compared to a healthy population.

### Urinalysis

**FIGURE 1:**
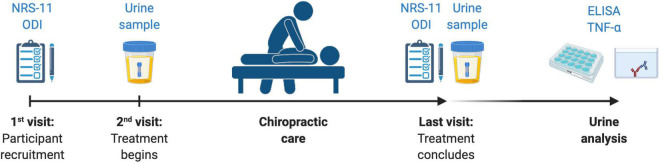
The study protocol, including outcomes measures collected in relationship to treatment period. NRS-11, Numerical Rating Scale 11; ODI, Oswestry Disability Index; TNF-α, Tumor Necrosis Factor-alpha.

Both patients and healthy participants provided an initial urine sample (first micturition of the morning) before any treatment was initiated. Control subjects provided a unique urine sample at the beginning of the study as the only outcome measure, since they did not receive any treatment and the levels of TNF-α do not seem to change significantly over time in the absence of active or chronic inflammation (Feghali and Wright, 1997; Biancotto et al., 2013; Wang et al., 2016). Patients collected their first urine sample on the same morning of the initial treatment session and a urine sample on the morning after the last session of the treatment period (Figure 1). For all cases, patients were instructed to provide the first micturition of the morning and preserve their sample refrigerated for as long as possible before their appointment in the Clinic. Appointments with urine sample collection were scheduled first thing in the morning. Urine samples were then collected from patients and immediately stored in a container at −20°C. Urine concentration of TNF-α was measured in duplicate by using specific commercial sandwich enzyme-linked immunosorbent assay (ELISA) following manufacturer’s recommendations (Cloud-Clone Corp., TX, United States) (Sirera et al., 2003). For each sample, the urinary concentrations of TNF-α (pg/ml) and creatinine (mg/dl) were assessed. The ratio of urinary TNF-α to urinary creatinine in pg/mg was calculated to correct changes in urine volume (Ortega et al., 2019).

### Patient-Reported Outcome Measures

Patient-reported outcome measures (PROMs) were used to evaluate pain and disability. Both were measured at the beginning and upon completing the treatment period (Figure 1). Patients reported current pain intensity verbally using a Numerical Rating Scale from 0 to 10 (NRS-11), in which 0 signified the absence of pain and 10 the worst possible pain for the patient. Functional disability due to CLBP was measured using the validated version in Spanish of the Oswestry Disability Index (ODI) (Alcántara-Bumbiedro et al., 2006). The ODI questionnaire consists of 10 multiple-choice questions rated from 0 to 5, with a total possible score of 50 (maximal disability).

### Statistical Analysis

In order to detect possible differences in normalized TNF-α (TNF-α to creatinine ratio) urinary concentrations between patients with CLBP and healthy controls, the mean value was calculated from the latter and used as a reference value. Further, Welch’s *t*-tests were used to compare the mean of the CLBP group to this reference value, both pre- and post-treatment. This adaptation of the *t*-test is known to be robust against type I errors (Derrick et al., 2016). Urinary TNF-α concentrations before and after chiropractic treatment were later compared by using a paired *t*-test. Additionally, paired *t*-tests were used to ascertain differences in PROMs (NRS-11 and ODI scores) before and after exposure to chiropractic treatment. In order to assess for correlations between concentrations of TNF-α, pain intensity, disability and number of visits, Spearman’s rank correlation coefficient was calculated by using before and after treatment values and percent changes. Finally, the potential interaction of treatment variables other than SMT (exercise and medication use) was analyzed by comparing the mean variations in TNF-α levels (Δ_TNF–α_), NRS-11 (Δ_NRS–11_) and ODI scores (Δ_ODI_) in participants following a home exercise program or using pain medication, versus participants who did not. Differences in these variables were based on pragmatic differences in the treatment approach. Due to unequal sample sizes and variances, Welch’s *t*-test were used for these comparisons. Values presented in the results section represent mean ± standard deviation. For all before and after comparisons, effects sizes were computed and reported by means of Cohen’s *d*. A *p* value threshold of 0.05 was considered statistically significant for all analyses.

## Results

Twenty-four patients (14 men and 10 women) CLBP met the inclusion and exclusion criteria, accepted to participate in the study by providing PROMs and urine samples, and concluded the treatment period. All participants included in the study were new patients to the Clinic and had never received chiropractic care prior to the study. Four additional patients were initially recruited but after further scrutiny, 2 of them did meet one of the exclusion criteria (suspected inflammatory spondyloarthropathy, and diagnosis of depression) and another 2 did not complete the treatment period, thus, their data were excluded from the analyses. Treatment consisted of a mean of 8.4 individual visits (ranging from 3 to 13 visits), delivered during a mean of 39.4 days (ranging from 19 to 76 days). As far as the control group is concerned, 5 healthy subjects (3 men and 2 women) were recruited for the study. Table 2 provides a summary of the participants demographic and baseline information.

**TABLE 2:** Participant demographic and baseline data.

	Chronic low back pain sample	Control sample
Sample n	24	5
**Sex, n (proportion)**		
Women	10 (41.7%)	2 (40%)
Men	14 (58.3%)	3 (60%)
**Age, mean ± SD (range)**		
Total sample	53.2 ± 12.1 (26–75)	44.9 ± 8.3 (39–60)
Women	54.6 ± 8.2 (45–62)	40.5 ± 2.1 (39–42)
Men	52.2 ± 14.6 (26–75)	59.3 ± 9.5 (42–60)
**Urinary TNF-α and Creatinine baseline concentrations, mean ± SD (range)**		
Baseline TNF-α concentration (pg/ml)	7.9 ± 11.3 (0–44.1)	0.4 ± 0.9 (0–2.0)
Baseline creatinine concentration (mg/dl)	172.9 ± 115.2 (21.7–469.9)	132.5 ± 80.0 (38.5–244.9)
Baseline TNF-α to ceatinine ratio (pg/mg)	6.0 ± 7.0 (0–21.3)	0.4 ± 0.8 (0–1.8)
**Chronic low back pain patients’ profile**		
Pain duration, years ± SD (range)	7.8 ± 8.9 (0.5–24)	–
Episodic pain, n (proportion)	6 (25%)	–
Ongoing pain, n (proportion)	18 (75%)	–
**Treatment**		
Duration of chiropractic care, days ± SD (range)	39.4 ± 17.4 (19–76)	–
Treatment sessions, number ± SD (range)	8.4 ± 3.5 (3–13)	–

### Urinary Levels of Tumor Necrosis Factor-Alpha in Healthy Participants

The mean concentration of TNF-α in urinary samples of healthy participants was found to be 0.4 ± 0.9 pg/ml, and when corrected for urinary volume, the ratio to creatinine was 0.4 ± 0.8 pg/mg (see Table 2).

### Urinary Levels of Tumor Necrosis Factor-Alpha in Chronic LBP Patients

Before initiating care, mean urinary TNF-α concentration was 7.9 ± 11.3 pg/ml and the mean ratio to creatinine was 6.0 ± 7.0 pg/mg (see Table 2). These levels were significantly higher when compared to the reference value obtained from healthy controls (*p* = 0.0006).

After completing the period of chiropractic care, the mean concentration of TNF-α in urine was 3.6 ± 5.5 pg/ml, and the ratio to creatinine was 2.8 ± 4.5 pg/mg. These levels were still significantly elevated when compared to our reference value (*p* = 0.015).

### Changes in Urinary Levels of Tumor Necrosis Factor-Alpha

**FIGURE 2:**
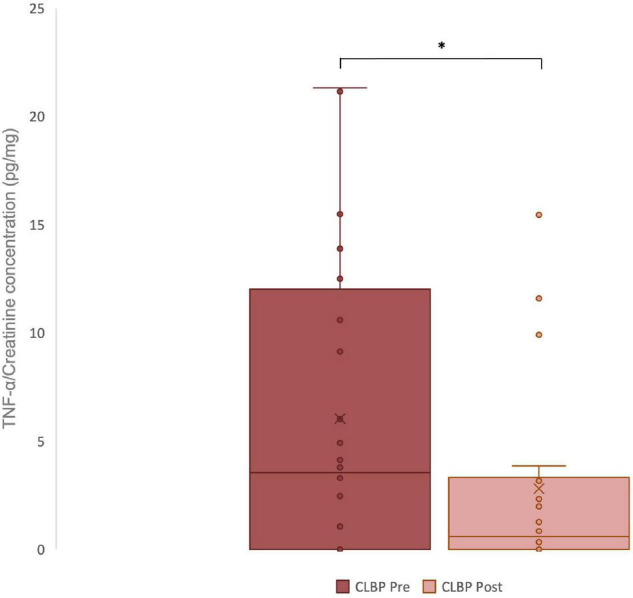
Urinary TNF-α levels in chronic low back pain (CLBP) patients, pre- and post-treatment. For each sample, the urinary concentrations of TNF-α (pg/ml) and creatinine (mg/dl) were assessed. The ratio of urinary TNF-α to urinary creatinine in pg/mg was calculated to correct changes in urine volume. The middle line represents the median and the *x* represents the mean. The upper and the lower lines of the box represent the first and third quartile respectively and the whiskers include all individual data points within 1.5 times the interquartile range. *p < 0,05.

Urinary concentration of TNF-α corrected for volume was significantly lower after the period of chiropractic care compared to baseline (*p* = 0.03), Cohen’s *d* = 0.55. These results are illustrated in Figure 2.

### Changes in Pain Intensity and Disability

**FIGURE 3:**
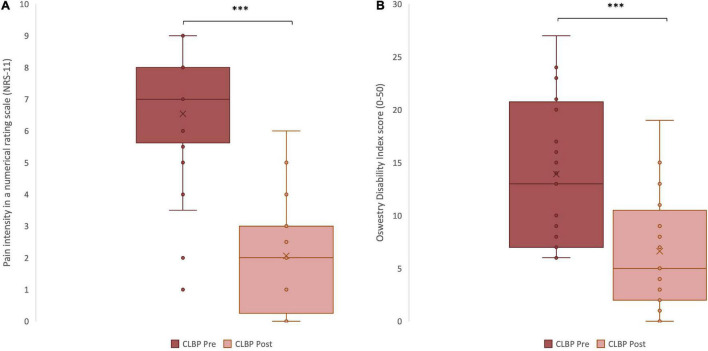
Clinical variables measured in chronic low back pain (CLBP) patients, pre- and post-treatment. **(A)** Pain intensity scores reported in a Numerical Rating Scale, from 0 to 10, pre- and post- treatment ^***^p < 0.001. **(B)** Disability scores reported in the Oswestry Disability Index questionnaire, from 0 to 50, pre- and post-treatment. The middle line represents the median and the *x* represents the mean. The upper and the lower lines of the box represent the first and third quartile respectively and the whiskers include all individual data points within 1.5 times the interquartile range. ^***^p < 0.001.

Following the period of chiropractic care a statistically significant reduction in pain intensity (as measured by a numerical rating scale: NRS-11) was noted for the treatment group, *p* < 0.001, Cohen’s *d* = 2.33 (Figure 3A). Moreover, upon completing the period of chiropractic treatment, a statistically significant reduction in functional disability (as measured by the Oswestry Disability Index: ODI) was observed, *p* < 0.001, Cohen’s *d* = 1.17 (Figure 3B).

Spearman’s rank coefficient highlighted a moderate significant correlation between NRS-11 and ODI scores before (ρ = 0.46, *p* = 0.01) and after treatment (ρ = 0.52, *p* = 0.005. The percentages of change in NRS-11 and ODI scores were also positively correlated (ρ = 0.55, *p* = 0.003). Of interest, there was also a moderate significant correlation between the number of visits and the change in NRS-11 (ρ = 0.49, *p* = 0.008). However, no other variable was correlated to the number of visits (change in ODI ρ = 0.27, *p* = 0.1; ODI post-treatment ρ = 0.33, *p* = 0.056; NRS-11 post-treatment ρ = 0.32, *p* = 0.06). No significant correlations were observed between TNF-α urinary levels at any stage or percent changes before and after treatment with any other variables analyzed (all *p*’s > 0.2).

### Effect of Other Treatments on Patient-Reported Outcomes and Tumor Necrosis Factor-Alpha

Out of 24 patients, seven received recommendations for a home exercise program apart from SMT. Patients following and not following a home exercise program showed similar reductions in TNF-α levels (*p* = 0.9), pain intensity (*p* = 0.3) and disability (*p* = 0.8). Table 3 provides the details of these comparisons.

**TABLE 3:** Before and after treatment variations in TNF-α concentrations, pain intensity and disability in patients following or not following a home exercise program, and using or not using pain medication during the study.

	Home exercise program (YES)	Home exercise program (NO)	*p*-value	Pain medication (YES)	Pain medication (NO)	*p*-value
Sub-sample n (proportion)	7 (29%)	17 (61%)	–	7 (29%)	17 (61%)	–
**Mean variations (Δ_Post–Pre_)**						
TNF-α concentrations	−3.48	−3.13	0.92	−1.91	−3.77	0.43
Pain intensity ratings	−3.64	−4.82	0.30	−4.71	−4.38	0.78
Oswestry disability index	−7.86	−7.06	0.80	−11.43	−5.59	0.11

*p-values obtained from comparing the means of both sub-samples using a Welch’s t-test.*

Seven patients were taking non-opioid analgesics, muscle relaxants or non-steroidal anti-inflammatory drugs (NSAIDs), one of them was also taking Pregabalin and another one Tramadol. No significant differences were observed between patients using pain medication and those who were not, for variations before and after treatment in TNF-α levels (*p* = 0.4), pain intensity (*p* = 0.8) and disability (*p* = 0.1) (Table 3).

## Discussion

To the best of our knowledge, this is the first study to measure TNF-α levels in urine samples of CLBP patients. In this cohort of 24 patients with CLBP, urinary concentrations of this cytokine were significantly elevated when compared to a reference value from healthy controls. Urinary TNF-α levels were lower after chiropractic care compared with the baseline values. This was accompanied by lower levels of pain and disability after chiropractic care compared with the levels before. However, clinical variables and TNF-α levels were not correlated. While this study cannot make any causal connection between changes in TNF-α and treatment, future studies with a control group should be able to determine whether chiropractic care may relieve pain and decrease disability in patients with CLBP through modulation of the pro-inflammatory cytokine network.

Although urinary levels of inflammatory cytokines have already been measured and highly correlate to serum levels, they have not been used specifically as biomarkers for chronic pain conditions (Moldawer, 1997; Sirera et al., 2003; Prasad et al., 2016). In this study, we detected elevated concentrations of TNF-α in urine samples of CLBP patients (both before and after treatment), when compared to our reference value, suggesting that urine could be a reliable milieu in which biomarkers for CLBP can be studied. After the chiropractic care period, TNF-α concentrations were significantly reduced, with a medium effect size (*d* = 0.55). Similarly, Wang et al. found a significant difference in the proportion of participants with elevated levels of serum TNF-α positive when comparing healthy subjects and CLBP patients before treatment (Wang et al., 2008). In this study, a multimodal treatment program of conservative care (not including SMT) diminished the initial proportion of TNF-α positive patients, although not reaching levels found in a healthy cohort (Wang et al., 2008).

Data on a potential influence of SMT on proinflammatory cytokines including TNF-α is still scarce. Teodorczyk-Injeyan et al. (2006) collected blood samples of asymptomatic subjects randomly assigned to either SMT, sham SMT or venipuncture control groups. Blood cultures were stimulated with lipopolysaccharide to induce *in vitro* production of proinflammatory cytokines for 24 h. The study showed a time-dependent significant attenuation of the *in vitro* production of inflammatory cytokines (including TNF-α) in the SMT group (Teodorczyk-Injeyan et al., 2006). More recently, the same group reported significantly higher *in vitro* TNF-α production in acute and chronic LBP patients compared to asymptomatic controls, and a significant reduction after 2 weeks of chiropractic care when compared to changes in the same controls (Teodorczyk-Injeyan et al., 2021). This effect of SMT has not been consistently reported throughout the literature. Degenhardt et al. (2017) did not find significant differences in cytokine levels (including TNF-α) when comparing CLBP patients to healthy controls, before or after receiving manual therapy. Probably, defining CLBP after 6 weeks and limiting treatment to one session may have influenced the results. Similarly, Song et al. (2016) were not able to identify any detectable changes in TNF-α, IL-1β and IL-10 plasma levels of rats with experimental neuropathic and postoperative pain receiving SMT sessions. Instead, elevated levels of the anti-inflammatory cytokine IL-10 were observed locally in the spinal cord. Further research is needed in this area to clarify the link between SMT and concentrations of both serum and urinary inflammatory markers.

The physiological mechanism through which SMT can result in a reduction of pro-inflammatory cytokines is still unknown. Recent studies have shown that activation of pain circuits regulates neuroinflammation, both centrally and in the periphery (Ji et al., 2016, 2018). It seems plausible that SMT-induced modulation of nociceptive input (Gevers-Montoro et al., 2021a) could result in a down-regulation of neuroinflammation. Such neuroimmune interactions have been extensively researched in the context of chronic pain, suggesting a bi-directional communication between nociceptive neurons and microglia necessary for the release of pro-inflammatory mediators such as TNF-α (Ji et al., 2016, 2018). Furthermore, central release of TNF-α seems to be required to induce long-term potentiation and central sensitization in the dorsal horn of the spinal cord, inducing persistent pain (Kawasaki et al., 2008; Ji et al., 2016, 2018). Similarly, in both animal models and patients with rheumatoid arthritis or discogenic LBP, TNF-α blocking drugs were shown not only to reverse central pain responses but also to improve disability (Hess et al., 2011; Sainoh et al., 2016).

On the other hand, it has also been suggested that SMT could have a non-specific effect on sympathetic nervous system (SNS) function (Wirth et al., 2019), which could in turn modulate immune responses (Teodorczyk-Injeyan et al., 2006). Involvement of the SNS in the modulation of TNF-α production and release by macrophages has been observed in animal models (Szelenyi et al., 2000), and clinical applications of this interaction have shown promising outcomes (Poyhonen-Alho et al., 2008). Evidence for regulation of cytokine levels by the SNS in chronic pain conditions is still on early stages (David Clark et al., 2018). Further research is needed in order to determine the mechanisms mediating the reduction in TNF-α levels apparently induced by SMT or other conservative interventions. Resolution of inflammation through an inhibition of pro-inflammatory cytokine activity is currently considered a new therapeutic frontier in the field of chronic pain (Ji et al., 2018).

Our findings are in line with previous data suggesting that SMT is an effective approach to reduce pain levels and improve function in CLBP patients (Rubinstein et al., 2019). The levels of pain intensity and disability were both significantly reduced after the treatment period, although these effects were not compared with a placebo. Nevertheless, the changes observed had very large effect sizes (*d* = 2.33 and 1.17 for pain and disability respectively), which could also be considered clinically significant. Indeed, pain intensity ratings showed a 68% reduction in pain intensity from baseline, largely exceeding the proposed threshold of 30% (Ostelo et al., 2008), while disability scores were 14.6 points lower (out of 100) after treatment, also superior to the estimated minimal detectable change for the ODI of 11.75 points (Johnsen et al., 2013). The present study identified moderate significant correlations between pain intensity levels and disability scores percentage of change, which is consistent with the ODI being an adequate tool to measure LBP-related disability.

On the other hand, no strong correlation was observed between TNF-α levels and NRS-11 and ODI scores, suggesting that the levels of pain and disability are not linearly related to the cytokine profile in our sample or that cytokine levels are affected by other factors. Changes after chiropractic treatment on one variable did not predict possible alterations of the others. This lack of correlation was also reported by Wang et al. (2008) and could be explained by an insufficient sample size. It has also been speculated that TNF-α may be associated with the lack of recovery from LBP, which may explain the lack of correlation with pain and disability (Morris et al., 2020). Nevertheless, other authors reported correlations between pain intensity and pro-inflammatory cytokines (including TNF-α) in plasma (Koch et al., 2007; Sikorska et al., 2019). Interestingly, TNF-α levels were found to be significantly augmented in patients with severe pain when compared to patients with milder pain intensity (Koch et al., 2007; Wang et al., 2016).

### Limitations of the Study

This study has several limitations, the main ones arising from the nature of the study itself. Being of observational nature, care was not standardized for all participants, and this includes type of care utilized, number of treatment sessions and length of the treatment period. Nevertheless, this approach has the advantage of offering a more ecological setting, more representative of the reality of chiropractic practice (Walker et al., 2011; Beliveau et al., 2017). The observations drawn from this type of studies generally provide guidance for the design of future clinical trials.

Another important limitation of the study lies in the method of urine sample collection and processing. In urine, factors such as timing of voids, and time spent at room temperature may affect the quality of recovered protein data (Siddiqui et al., 2015). Despite having informed participants on the proper method for urine sample collection, whether all participants followed the recommended steps cannot be guaranteed, which may introduce variability and affect the reproducibility of the data.

The common denominator in the study was always SMT, which was used in every patient for every visit. However, as beforementioned, exercise therapy exerts a beneficial influence over CLBP, partially based on modulating inflammatory cytokine profiles including TNF-α (Cheng et al., 2015; Leung et al., 2016; James et al., 2018a). The introduction of exercise therapy did not seem to provide additional benefit in our sample, although the sample size is too small to draw any conclusion. However, it is noteworthy to consider that future research should look at SMT and exercise independently from each other preferably.

Moreover, some patients were also taking pain medication of unknown posology (due to missing data and self-medication). In our cohort, medication use did not yield a significant effect on TNF-α levels or pain intensity, yet NSAIDs can alter cytokine levels, conducing to a measurement bias (Yan et al., 2018). Furthermore, some studies reported no effect of oral analgesics (not including anti-inflammatory drugs) on serum TNF-α levels (Wang et al., 2008) or even a spontaneous increase in production from peripheral blood monocytes and synovial membrane cultures after exposure to non-steroidal anti-inflammatory drugs (Page et al., 2010).

To confirm our preliminary findings would require conducting further research. It is not possible to conclude that any of the measured effects are the direct consequence of the care received, unless a second CLBP group is included that will be randomly assigned to receive a different intervention, a sham intervention or no intervention at all. Moreover, our control group for urinary TNF-α levels was small when compared to the CLBP group and only one measure was taken at the beginning of the protocol. A second measure at the end of the protocol would allow to measure and control for non-specific changes in TNF-α levels. More data is needed in order to explore the potential for urinary TNF-α and other cytokines to become biomarkers for CLBP that could be quantified in clinical practice.

## Conclusion

To our knowledge, this is the first study to suggest that urinary levels of TNF-α can be found elevated in patients with CLBP when compared to healthy individuals. The results obtained in this preliminary study suggest that a non-standardized period of chiropractic care could have a significant impact on pain and disability levels, as well as on urinary concentrations of pro-inflammatory cytokine TNF-α in CLBP patients. These potential benefits should be further investigated in a proper randomized controlled trial design, with at least one control group that will not receive chiropractic care. Therefore, results from the current study should be interpreted with caution. Further research to elucidate the mechanisms and role of different conservative interventions for CLBP is an essential upcoming step.

## Data Availability Statement

The raw data supporting the conclusions of this article will be made available by the authors, without undue reservation.

## Ethics Statement

The studies involving human participants were reviewed and approved by Madrid College of Chiropractic Research Ethics, Real Centro Universitario Escorial María Cristina. The patients/participants provided their written informed consent to participate in this study.

## Author Contributions

CG-M: conceptualization, methodology, investigation, formal analysis, and writing – original draft preparation. MR-S and LL: investigation, formal analysis and manuscript revision. FC-B: investigation, resources, and manuscript revision. DN: conceptualization and writing- review and editing. LÁ-G: conceptualization, supervision, and writing- review and editing. MP: supervision, funding acquisition, and writing- review and editing. AO-DM: conceptualization, methodology, funding acquisition, supervision, writing- review, and editing and approval of final version. All authors contributed to the article and approved the submitted version.

## Conflict of Interest

The authors declare that the research was conducted in the absence of any commercial or financial relationships that could be construed as a potential conflict of interest.

## Publisher’s Note

All claims expressed in this article are solely those of the authors and do not necessarily represent those of their affiliated organizations, or those of the publisher, the editors and the reviewers. Any product that may be evaluated in this article, or claim that may be made by its manufacturer, is not guaranteed or endorsed by the publisher.
